# Death of Mr. Sherwood

**Published:** 1837-10

**Authors:** 


### DEATH OF MR. SHERWOOD.

It is with sincere regret that we announce the death of Mr. Sherwood, the senior partner in the House of our very respectable and worthy Publishers in Paternoster row. This event took place on Thursday, the 7th September, at Mr. Sherwood's country house at Holloway, in consequence of a paralytic attack. Mr. Sherwood was in his sixty-first year, but possessed, up to the period of his illness, the vigour and activity of an earlier age. The great services which Mr. Sherwood rendered to medical literature in this country, by his enterprise as a publisher, well entitles him to a notice in this journal. It is to him that we are entirely indebted for the publication of the Cyclopædia of Practical Medicine, recently completed, as well as of the Cyclopædias of Anatomy and of Surgery, now in progress. Of these works the House in Paternoster Row is, we believe, sole proprietors. Owing to the great success of the Cyclopædia of Medicine, Mr. Sherwood was induced to engage in the publication of the others; and also to become the proprietor and publisher of various medical works to a large extent, and greatly to the benefit of the profession. Although connected with the British and Foreign Medical Review in no other way than as Publisher, it is but justice to Mr. Sherwood to state, that he watched over its interests with the same zeal as if he had been Proprietor: and the Editors have great pleasure in bearing testimony to the marked attention, activity, liberality, and kindness which he uniformly displayed, during the constant intercourse which they had with him from the commencement of the Cyclopædia of Medicine, in 1831, up to the period immediately preceding his death. In his private and domestic relations Mr. Sherwood was most exemplary: he was greatly respected and beloved; and he is regretted by his surviving family and friends as so good and kind-hearted a man deserved to be regretted.

---

